# GCRTcall: a transformer based basecaller for nanopore RNA sequencing enhanced by gated convolution and relative position embedding via joint loss training

**DOI:** 10.3389/fgene.2024.1443532

**Published:** 2024-11-22

**Authors:** Qingwen Li, Chen Sun, Daqian Wang, Jizhong Lou

**Affiliations:** ^1^ Key Laboratory of Epigenetic Regulation and Intervention, Center for Excellence in Biomacromolecules, Institute of Biophysics, Chinese Academy of Sciences, Beijing, China; ^2^ College of Life Sciences, University of Chinese Academy of Sciences, Beijing, China; ^3^ Beijing Polyseq Biotech Co., Ltd., Beijing, China

**Keywords:** basecaller, nanopore RNA sequencing, transformer, gated convolution, relative position embedding

## Abstract

Nanopore sequencing, renowned for its ability to sequence DNA and RNA directly with read lengths extending to several hundred kilobases or even megabases, holds significant promise in fields like transcriptomics and other omics studies. Despite its potential, the technology’s limited accuracy in base identification has restricted its widespread application. Although many algorithms have been developed to improve DNA decoding, advancements in RNA sequencing remain limited. Addressing this challenge, we introduce GCRTcall, a novel approach integrating Transformer architecture with gated convolutional networks and relative positional encoding for RNA sequencing signal decoding. Our evaluation demonstrates that GCRTcall achieves state-of-the-art performance in RNA basecalling.

## Introduction

Nanopore sequencing technology directly sequences single strands of DNA or RNA by detecting changes in electrical current as the molecules pass through nanopores, eliminating the need for PCR amplification. This technique enables rapid single-molecule sequencing with significantly increased read lengths, reaching hundreds of kilobases or even magabases. It holds immense potential in various omics sequencing studies such as genomics, transcriptomics, epigenomics, and proteomics (Amarasinghe et al., 2020; Garalde et al., 2018; Jain et al., 2022; Sun et al., 2020; Jain et al., 2018; Davenport et al., 2021; Quick et al., 2016; Wang et al., 2015; Faria et al., 2017; Yakovleva et al., 2022; Boykin et al., 2019; Zhao et al., 2022).

Despite its advantages, the accuracy of basecalling has emerged as a significant bottleneck, limiting further broader application of nanopore sequencing. Sequencing signals are influenced not only by individual nucleotides but also by neighboring bases, resulting in non-uniform translocation of the sequences and low signal-to-noise ratios measured in picoamperes (pA). These challenges make accurate basecalling in nanopore sequencing particularly difficult (Jain et al., 2022; Wick et al., 2019; Wang et al., 2021).

In recent years, several algorithms have been developed to improve the accuracy of nanopore sequencing signal decoding and methylation detection. Methods like Metrichor and Nanocall (David et al., 2017), which utilize Hidden Markov Models (HMM) (Niu et al., 2022), segment events in the current signal and calculate transition probabilities for basecalling. Other approaches, such as Chiron (Teng et al., 2018), Deepnano (Boža et al., 2017), and Guppy, leverage Recurrent Neural Network (RNN) architectures, while Causalcall (Zeng et al., 2019) and RODAN (Neumann et al., 2022) employ Convolutional Neural Network (CNN) architectures to achieve end-to-end basecalling. Additionally, SACall incorporates self-attention mechanisms into nanopore signal decoding (Huang et al., 2022). Liu Q. et al. (2019) proposed DeepMod based on bidirectional long short-term memory (LSTM) (Chen et al., 2022) architecture to detect DNA modifications. Ni et al. (2019) developed DeepSignal by combining LSTM and Inception structure for DNA methylation prediction. Yin et al. (2024) constructed NanoCon through Transformer and contrastive learning for DNA modification detection. However, with the exception of RODAN (Neumann et al., 2022), the focus of these methods is primarily on DNA basecalling and modification prediction, with limited research in RNA decoding.

Unlike several hundreds base pairs per second (bps) translocation speed for DNA, RNA translocates at only about or below 100 bps. Additionally, there are substantial differences in the physical and chemical properties between DNA and RNA, resulting in distinct signal patterns. Consequently, models designed for DNA basecalling are usually ineffective for RNA signal decoding. To address this gap, we propose GCRTcall, a Transformer based basecaller for nanopore RNA sequencing, enhanced by Gated Convolution and Relative position embedding through joint loss training. This method achieves state-of-the-art decoding accuracy on multi-species transcriptome sequencing data.

## Materials and methods

### Benchmark dataset

The benchmark dataset used in this study was proposed by Neumann et al. (Neumann et al., 2022), which is also utilized in the development of RODAN (Neumann et al., 2022).

The training set comprises five species: *Arabidopsis thaliana* from (Neumann et al., 2022), Epinano synthetic constructs from (Liu H. et al., 2019), *Homo sapiens* from (Workman et al., 2019), *Caenorhabditis elegans* from (Roach et al., 2020), and *Escherichia coli* from (Grünberger et al., 2019). Initially, all reads were basecalled using Guppy version 6.2.1 (Technologies and O.N., 2024). The decoded sequences were then mapped to the reference genome with minimap2 (Li, 2018) to obtain corrected sequences. Subsequently, Taiyaki (Technologies and O.N., 2019) was utilized to generate an HDF5 file containing the raw signal of each read, its corresponding corrected sequence, and their mapping relationship. The training dataset contained 116,072 reads: with 24,370 from Arabidopsis, 29,728 from Epinano synthetic constructs, 30,048 from *H. sapiens*, 24,192 from *C. elegans*, and 7,734 from *E. coli*.

To ensure rigorous performance evaluation and avoid potential biases from overlapping datasets, we used test samples derived from entirely independent studies, distinct from those used for training. The test set included also five species: *H. sapiens* from (Workman et al., 2019), *A. thaliana* from (Parker et al., 2020), *Mus musculus* from (Bilska et al., 2019), *S. cerevisiae* from (Jenjaroenpun et al., 2021), and Populus trichocarpa from (Gao et al., 2021), each consisting of 100,000 reads.

### Model architecture

**FIGURE 1:**
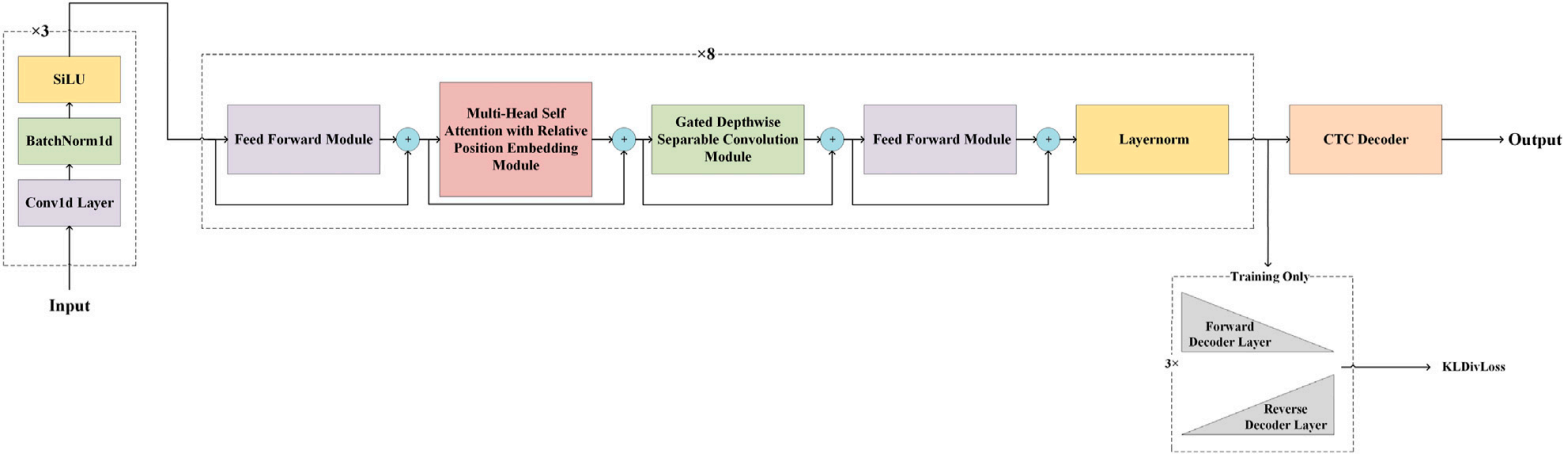
Schematics representation of the architecture of GCRTcall. GCRTcall compreises three CNN layers for downsampling and feature extraction, and followed by 8 Conformer blocks and a CTC decoder. During training, a pair of forward and reverse decoder was added on top of the base architecture for joint loss training.

Our model architecture was inspired by Google’s Conformer (Gulati et al., 2020), a convolution-augmented Transformer known for effectively modeling both global and local dependencies, outperforming traditional Transformer (Zhang et al., 2020; Vaswani et al., 2017; Liu B. et al., 2019; Zhang et al., 2024) and CNN (Li et al., 2019; Kriman et al., 2019; Han et al., 2020; Abdel-Hamid et al., 2014; Li et al., 2021; Li and Liu, 2023; Li X. et al., 2024) models in speech recognition tasks. GCRTcall compreises three CNN layers for downsampling and feature extraction, with output channels of 4, 6, and 512, and convolutional kernels of size 5, 5, and 19, with strides of 1, 1, and 10, respectively. This is followed by 8 Conformer blocks and a connectionist temporal classification (CTC) decoder (Gulati et al., 2020; Wang et al., 2023; Zhu et al., 2023), amounting to a total of 50 million parameters. Our previous study indicated that training with a joint loss, combining CTC loss and Kullback-Leibler Divergence (KLDiv) loss, results in superior basecalling accuracy compared to using only CTC loss under the same inference structure, and whether using decoder do not influence decoding accuracy. However, retaining the decoder results in a significant decrease in inference speed (Li Q. et al., 2024). Therefore, during the training phase, GCRTcall incorporates additional forward and backward Transformer decoders at the top, utilizing the joint loss for improved convergence. The model architecture of GCRTcall is illustrated in Figure 1.

Compared to traditional Transformers, the Conformer modules in GCRTcall feature two key improvements: First, they combine relative positional embedding with a multi-head self-attention mechanism to enhance the model’s robustness to inputs of varying lengths. Second, they integrate depthwise separable convolutions based on gate mechanisms to process the outputs of attention layers, thereby strengthening the model’s ability to capture local dependencies within sequences.

### Relative position multi-head self-attention mechanism

Transformer-XL (Dai et al., 2019) integrates relative positional embedding with a self-attention mechanism, enhancing the model’s representational capacity for sequences of varying lengths. The relative position multi-head self-attention mechanism processes input sequences along with its sinusoidal position encoding. It performs three linear projections on the input to generate *Q, K*, and *V*, and also applying linear projection on positional embedding to obtain *K*p. Two biases, *bk* and *bp*, are initialized. The computation principle of the relative position self-attention mechanism is as follows:

$$\mathrm{RelativeAttention}=\mathrm{softmax}\left(\frac{\left(Q+bk\right){\times K}^{T}+relative\_shift\left[\left(Q+bp\right){\times Kp}^{T}\right]}{\sqrt{d_{k}}}\right)V$$

The multi-head attention then combines and projects the aforementioned attention computation results as follows:

$$\mathrm{MultiHead}=\mathrm{Concat}\left({\mathrm{head}}_{1},...,{\mathrm{head}}_{h}\right)W^{O}$$

### Gated depthwise separable convolution

EfficientNet (Tan et al., 2019) utilizes depthwise separable convolution to reduce the number of parameters and enhance computational efficiency while maintaining state-of-the-art accuracy. Similarly, Dauphin et al. (Dauphin et al., 2016) proposed gated convolutional networks, which utilize CNNs to extract hidden states from sequences and employ gated linear units (GLU) to augment non-linear expression and mitigate the vanishing gradient problem. This approach enables the model to compute in parallel, outperforming LSTMs on multiple NLP datasets. GLU is computed as follows:

$$\mathrm{GLU}\left(a,b\right)=a⊗\sigma \left(b\right)$$

where a is the first half of the input matrices and b is the second half.

**FIGURE 2:**
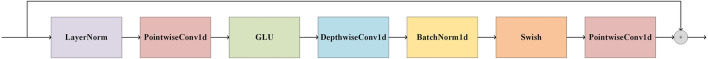
Architecture of the gated depthwise separable convolution block of GCRTcall. The convolution block consists of a 1-D pointwise convolution followed by a GLU, a 1-D depthwise convolution, 1-D batch normalization, and a swish activation function.

Inspired by these approaches, the structure of the gated depthwise separable convolution block in GCRTcall is illustrated in Figure 2.

### Joint loss training

An additional forward and reverse transformer decoder were added on the top of the inference structure of the model during training. The forward decoder adopts a lower triangular matrix as a causal mask, while the reverse decoder is equipped with an anti-lower triangular causal mask.

The model is trained by optimizing a joint loss that includes CTC loss and KLDiv loss to ensure convergence.

$$L_{\text{joint}}\left(x,y\right)=\lambda L_{\text{CTC}}\left(x_{E},y\right)+\left(1-\lambda \right)L_{\text{KLDiv}\;}\left(x_{D},y\right)$$

where *x*_E_ is the output probability matrix of the encoder, and *x*_D_ is the output of the decoders, *y* is the label, *λ* is a hyperparameter between 0 and 1. In this paper, *λ* was set to 0.5.

### Model training

As previously demonstrated, using a joint loss that combines CTC loss and KLdiv loss can help accelerate model convergence (Li Q. et al., 2024). Therefore, during training, we added two layers of forward and backward Transformer decoders at the top of GCRTcall, which are not utilized during actual inference.

GCRTcall was trained on an Ubuntu server equipped with 2 × 2.10 GHz 36-core CPUS, providing 144 logical CPUs and 512 GB of RAM. The training utilized 2 NVIDIA RTX 6000 Ada Generation 48G GPUs for 12 epochs, totaling 12.95 h. The batch size of 140 was employed, managed by the Ranger optimizer at a learning rate of 0.002 and weight decay of 0.01. The training was conducted using the ReduceLROnPlateau learning rate scheduler based on validation set loss monitoring with patience of 1, factor of 0.5, and threshold 0f 0.1.

### Performance evaluation

Identity, mismatch rate, insertion rate, and deletion rate were adopted as metrics to evaluate the decoding accuracy of the model. These overall median metrics are commonly used in multiple basecaller researches for performance evaluation and comparison:

$$Identity=\frac{Number\;of\;matched\;bases}{Length\;of\;alignment}\times 100\%$$

$$Mismatch\;rate=\frac{Number\;of\;mismatched\;bases}{Length\;of\;alignment}\times 100\%$$

$$Insertion\;rate=\frac{Number\;of\;inserted\;bases}{Length\;of\;alignment}\times 100\%$$

$$Deletion\;rate=\frac{Number\;of\;deleted\;bases}{Length\;of\;alignment}\times 100\%$$

## Results and discussion

### Comparison of decoding performance with different basecallers

**FIGURE 3:**
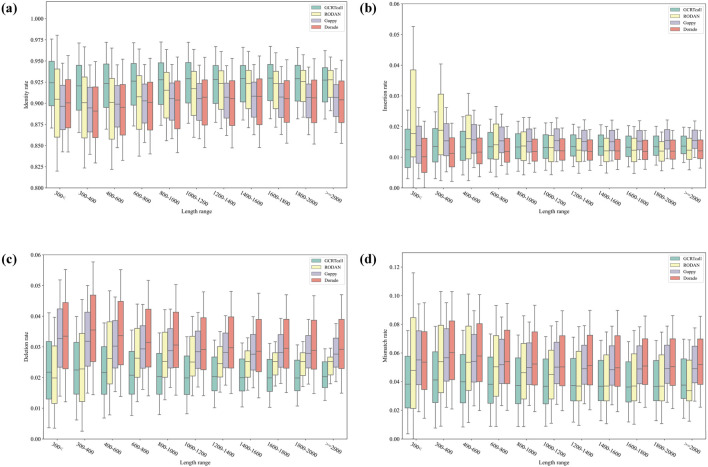
Performance comparison of different basecallers at different decoding lengths. **(A)** Identity rate comparison of different basecallers under different sequence lengths. **(B)** Insertion rate comparison of different basecallers under different sequence lengths. **(C)** Deletion rate comparison of different basecallers under different sequence lengths. **(D)** Mismatch rate comparison of different basecallers under different sequence lengths.

We compared the basecalling accuracy of GCRTcall, Guppy 6.2.1, Dorado 0.8.1, and RODAN on a test set consisting of five species. All basecalling results were aligned to the reference genomes using minimap2, retaining only the optimal alignment results. As shown in Table 1, GCRTcall achieved state-of-the-art accuracy levels across all five species. Notably, according to Neumann et al. (Neumann et al., 2022), while RODAN slightly outperforms Guppy in basecalling accuracy for mouse and yeast, GCRTcall significantly outperforms both in decoding accuracy for these two species. Additionally, all four basecallers exhibited the poorest performance on mouse, consistent with previous findings that suggest substantial differences in sequencing signal patterns between mice and other species. Further, the performance of various basecallers was compared at different decoding lengths (Figure 3). It can be seen that GCRTcall performs best across all lengths, especially in the case of extremely long read length, GCRTcall still maintains the leading decoding accuracy.

**TABLE 1:** Performance comparison between GCRTcall, RODAN, Dorado, and Guppy.

Species	Basecaller	Identity (%)	Insertion rate (%)	Deletion rate (%)	Mismatch rate (%)
Yeast	Guppy	91.36	1.57	2.48	4.56
	Dorado	92.08	1.31	2.34	4.23
	RODAN	90.86	1.67	2.52	5.17
	GCRTcall	**92.59**	**1.54**	**2.02**	**3.83**
Human	Guppy	90.59	1.42	3.02	4.89
	Dorado	88.77	1.15	3.86	6.11
	RODAN	92.75	1.27	2.09	3.69
	GCRTcall	**94.22**	**1.10**	**1.68**	**2.85**
Mouse	Guppy	87.67	1.73	3.70	6.84
	Dorado	85.88	1.10	4.72	8.23
	RODAN	86.82	**1.41**	3.97	7.36
	GCRTcall	**89.96**	1.44	**3.07**	**5.44**
Poplar	Guppy	90.15	1.66	2.81	5.31
	Dorado	91.36	1.32	2.57	4.66
	RODAN	90.55	**1.49**	2.53	5.36
	GCRTcall	**92.25**	1.51	**2.07**	**4.08**
Arabidopsis	Guppy	91.58	1.33	2.69	4.36
	Dorado	91.84	1.03	2.77	4.31
	RODAN	93.93	**1.10**	1.87	2.63
	GCRTcall	**94.03**	1.14	**1.77**	**2.94**

The bold values represent the evaluation metrics for the basecaller that performed the best under the respective test criteria for the species in question.

The inference was conducted on an Ubuntu server equipped with an Intel i9-13900K CPU, 125G RAM, and one NVIDIA RTX 3090 24G GPU. The inference speed of different basecaller model was also evaluated and compared. Dorado achieved the fastest decoding speed at 4.86E+07 samples per second because of its highly industrial optimization. Guppy reached decoding speed at 1.02E+07 samples per second, owing to its smaller parameter count of 2.2M. RODAN followed at 4.68E+06 samples per second, while GCRTcall, with its 50M parameters, completed decoding with speed at 1.68E+06 samples per second. Several acceleration optimization algorithms for Transformer-based models, such as hardware-aware techniques, sparse attention, and model quantization, have been proposed to enhance inference speed. These algorithms will be tested in the future development of GCRTcall.

In addition, we compared the decoding performance of Guppy, Dorado, and GCRTcall on RNA004 sample of Hek293T from (Chen et al., 2021), as presented in Table 2. Since the GCRTcall model was trained on the RNA002 dataset, and RNA004 differs significantly from RNA002 in terms of signal characteristics and sequence composition, GCRTcall’s performance on RNA004 is understandably inferior to that of Guppy and Dorado. The distinct signal features and species-specific differences in RNA004 require stronger generalization capabilities from GCRTcall, which was not trained on these new data patterns, leading to a decline in performance.

**TABLE 2:** Performance comparison between Dorado, Guppy, and GCRTcall on RNA004 sample.

	Identity (%)	Insertion rate (%)	Deletion rate (%)	Mismatch rate (%)
Dorado	96.54%	0.54%	0.94%	1.95%
Guppy	96.52%	0.54%	0.95%	1.97%
GCRTcall	81.48%	2.14%	5.27%	10.97%

In contrast, both Guppy and Dorado have been optimized with profiles specifically tailored for RNA004 sequencing data, enabling them to better adapt to RNA004 and its corresponding human cell line data. While GCRTcall performs well on RNA002, the performance discrepancy on RNA004 highlights limitations in the model’s generalization abilities across different RNA sequencing datasets.

To enhance GCRTcall’s performance on RNA004 and other emerging datasets, we plan to expand the model’s training set in future work to include more diverse RNA data sources, particularly sequencing data from RNA004 and other human cell lines. By incorporating broader datasets, we expect a significant improvement in the model’s generalization capabilities. Additionally, we aim to explore fine-tuning strategies specifically tailored for different RNA datasets to better address the challenges posed by varying signal patterns.

In future studies, we will focus on enhancing the model’s decoding accuracy, particularly in handling novel RNA sequencing data and more complex signal patterns. By integrating larger, more diverse datasets with continuous architectural optimizations, we expect that GCRTcall will achieve more stable and efficient performance across a wider range of transcriptomic applications.

### Ablation study

To further explore the impact of model structures on the basecalling accuracy of GCRTcall, we conducted two sets of ablation experiments: first, removing relative shift operation for position scores (GCRTcall w/o RS); and second, replacing Conformer modules with Transformer modules (Transcall).

In Transformer-XL, absolute position representation is initially performed to reduce the computational complexity of relative positional encoding. A relative shift of position scores is then applied to obtain relative position embeddings for sequences. To investigate the impact of relative position embeddings on model performance, GCRTcall was trained without the relative shift operation for 12 epochs using the same training set. The test results (Table 3) show a decrease in decoding performance compared to GCRTcall, indicating that relative position embeddings enhance the robustness of attention mechanisms to sequence position representation.

**TABLE 3:** Performance comparison between GCRTcall, GCRTcall w/o RS, and Transcall.

Species	Basecaller	Identity (%)	Insertion rate (%)	Deletion rate (%)	Mismatch rate (%)
Yeast	Transcall	90.07	1.83	2.98	5.07
	GCRTcall w/o RS	91.54	1.65	2.44	4.30
	GCRTcall	**92.59**	**1.54**	**2.02**	**3.83**
Human	Transcall	91.24	1.39	2.97	4.28
	GCRTcall w/o RS	93.03	1.33	2.02	3.38
	GCRTcall	**94.22**	**1.10**	**1.68**	**2.85**
Mouse	Transcall	87.02	1.49	4.46	6.93
	GCRTcall w/o RS	88.61	1.61	3.57	6.11
	GCRTcall	**89.96**	**1.44**	**3.07**	**5.44**
Poplar	Transcall	89.71	1.76	3.14	5.30
	GCRTcall w/o RS	91.06	1.69	2.47	4.62
	GCRTcall	**92.25**	**1.51**	**2.07**	**4.08**
Arabidopsis	Transcall	91.24	1.40	2.97	4.28
	GCRTcall w/o RS	92.87	1.31	2.19	3.45
	GCRTcall	**94.03**	**1.14**	**1.77**	**2.94**

The bold values represent the evaluation metrics for the basecaller that performed the best under the respective test criteria for the species in question.

To investigate the impact of gated convolution neural networks on model performance, Transformer modules were used to replace Conformer modules, and the model was also trained for 12 epochs. The test results (Table 3) indicate that the model’s decoding performance deteriorated compared to GCRTcall and GCRTcall w/o RS. This suggests that gated convolutional networks, which enhance the representation of local dependencies, play an important role in accurate basecalling.

**FIGURE 4:**
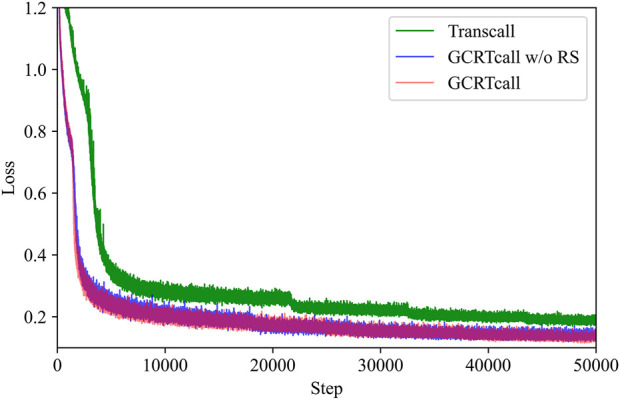
Training curves of GCRTcall, GCRTcall w/o RS, and Transcall. GCRTcall and GCRTcall w/o RS exhibit similar training curve. While Transcall, without convolutional enhancement, converges slower and to a higher loss compared to both GCRTcall and GCRTcall w/o RS.

The training curves of GCRTcall, GCRTcall w/o RS, and Transcall are illustrated in Figure 4. It can be observed that the form of position encoding has little impact on convergence during training, mainly enhancing the model’s generalization ability for decoding sequences of varying lengths. However, Transcall, without convolutional enhancement, converges slower and to a higher loss compared to both GCRTcall and GCRTcall w/o RS.

CNN is proficient at capturing local features due to their ability to apply convolutional filters across input sequences (Xiang et al., 2023). By integrating convolutional modules within each encoder layer, the model can effectively capture local patterns and features intrinsic to sequential data. This is crucial for recognizing local dependencies. Furthermore, convolutional operations can capture information at various scales by utilizing different kernel sizes. This allows model to integrate multi-scale contextual information, enhancing its representational capacity across different temporal granularities. The combination of self-attention and convolution allows the model to capitalize on the complementary strengths of self-attention and convolution. While self-attention mechanisms are adept at capturing global dependencies and long-range relationships, convolutional operations excel at extracting local features. This combination enables the model to handle both local and global contextual information efficiently.

Relative positional embedding captures the relative positional relationships between elements in a sequence, as opposed to absolute positional encoding which only indicates the position of each element. This approach is particularly beneficial for handling sequences of varying lengths, as it remains invariant to the length of the input sequence, thereby improving the model’s robustness. By using relative positional embedding, the model can flexibly represent positional information, which is crucial for tasks that rely heavily on the sequential nature of data, such as nanopore signal decoding. This encoding method allows the model to maintain a consistent representation of positional relationships, improving its ability to model sequences effectively.

## Conclusion

This study introduces GCRTcall, a Transformer-based basecaller designed for nanopore RNA sequencing signal decoding. GCRTcall is trained using a joint loss approach and is enhanced with gated depthwise separable convolution and relative position embeddings. Our experiments demonstrate that GCRTcall achieves state-of-the-art performance in nanopore RNA sequencing signal basecalling, outperforming existing methods in terms of accuracy and robustness. These results highlight the effectiveness of integrating advanced transformer architectures with convolutional enhancements for improving RNA sequencing accuracy.

Overall, GCRTcall represents a step forward in nanopore RNA sequencing, offering a robust and precise solution that can facilitate a deeper understanding of transcriptomics and other related fields.

## Data Availability

The original contributions presented in the study are included in the article/supplementary material, further inquiries can be directed to the corresponding authors.
